# Newborn With Bilateral Wrist Drop: A Rare Presentation of Isolated Radial Nerve Palsy

**DOI:** 10.7759/cureus.86879

**Published:** 2025-06-27

**Authors:** Ivone C Rodrigues, Daniela S Teixeira, Bruno D Paiva, Ida M Frazoa, Ana L Gomes

**Affiliations:** 1 Physical Medicine and Rehabilitation, Centro Hospitalar Universitário do Algarve, Faro, PRT

**Keywords:** bilateral wrist drop, newborn, peripheral nerve injury, radial nerve palsy, subcutaneous adipose necrosis

## Abstract

Isolated radial nerve palsy in newborns is rare, marked by impaired wrist and finger extension with preserved shoulder and elbow function. Differential diagnosis includes brachial plexus injuries and rare tumors. We present a case of a preterm infant, delivered by cesarean section after 35 weeks+5 days of uneventful pregnancy, who presented with bilateral wrist drop and asymmetrical skin lesions. Ultrasound revealed subcutaneous adipose necrosis in the left arm, suggesting radial nerve compression. Conservative management was initiated, focusing on passive mobilization and neurosensory stimulation. Over time, we observed a gradual recovery of wrist and finger extension bilaterally. Isolated radial nerve palsy in newborns usually presents a favorable prognosis, with spontaneous recovery typically occurring within six months. The early identification and differentiation of less serious conditions from more severe ones are crucial for effective management and for reassuring parents. Implementing early rehabilitation interventions, as demonstrated in the present case, can enhance recovery outcomes and mitigate the risk of musculoskeletal complications.

## Introduction

Bilateral radial nerve palsy in newborns is rare and often attributed to intrauterine compression or trauma during delivery, such as prolonged labor or difficult passage through the birth canal [1-3]. It can also be associated with conditions such as subcutaneous fat necrosis, which suggests underlying nerve compression [1,4,5]. Rarely, benign tumors such as angioleiomyomas or myofibromas can cause radial nerve palsy by compressing the nerve. These tumors are more commonly seen in adults but can occur in newborns, leading to nerve compression and requiring surgical intervention if severe [6].

The differential diagnosis should also include local bacterial infections (e.g., septic arthritis, osteomyelitis, or an abscess around the glenohumeral joint or shoulder region), as well as skeletal injuries such as humeral or clavicle fractures, since these conditions can lead to pseudoparalysis [7,8].

Radial nerve palsy may often be misdiagnosed as brachial plexus palsy due to a lack of awareness [1,3,5,6]. The incidence of neonatal brachial plexus injury is reported to be between 0.4 and 2.5 cases per 1,000 newborns [3]. On the other hand, cases of isolated radial nerve palsy in newborns are uncommon, predominantly affecting one side, with few cases being bilateral [1,7,8]. It is essential to distinguish between these two clinical conditions, as their treatment approaches and prognosis differ [5,7,9].

Radial nerve palsy is often diagnosed based on clinical examination, with symptoms including wrist drop and weakness in wrist and finger extensors, while proximal arm muscles remain unaffected [1-3,5,7]. Diagnosis is further supported by imaging studies, such as ultrasound and MRI, while electromyography confirms the involvement of the radial nerve [3,5].

Prompt diagnosis is essential to prevent long-term complications such as flexion contractures of the wrist and digits [3,4]. Clinical signs such as absent wrist and digital extension with intact shoulder and elbow function should raise suspicion of radial nerve palsy [1,3,4,7].

Conservative treatments, such as wrist splinting [3-5,7], taping [7], occupational therapy, and physiotherapy [3-5,7], have been found to be effective.

The prognosis for bilateral radial nerve palsy in newborns is generally excellent, with most cases showing complete recovery within three to six months without the need for surgical intervention [1,2,7,8].

## Case presentation

We present the case of a neonate born following a second, uneventful, monitored pregnancy lasting 35 weeks+5 days. Prenatal ultrasounds showed no alterations, and there was no reference to oligohydramnios. Delivery occurred by cesarean section due to stationary labor and rupture of membranes lasting more than 46 hours.

**Figure 1:**
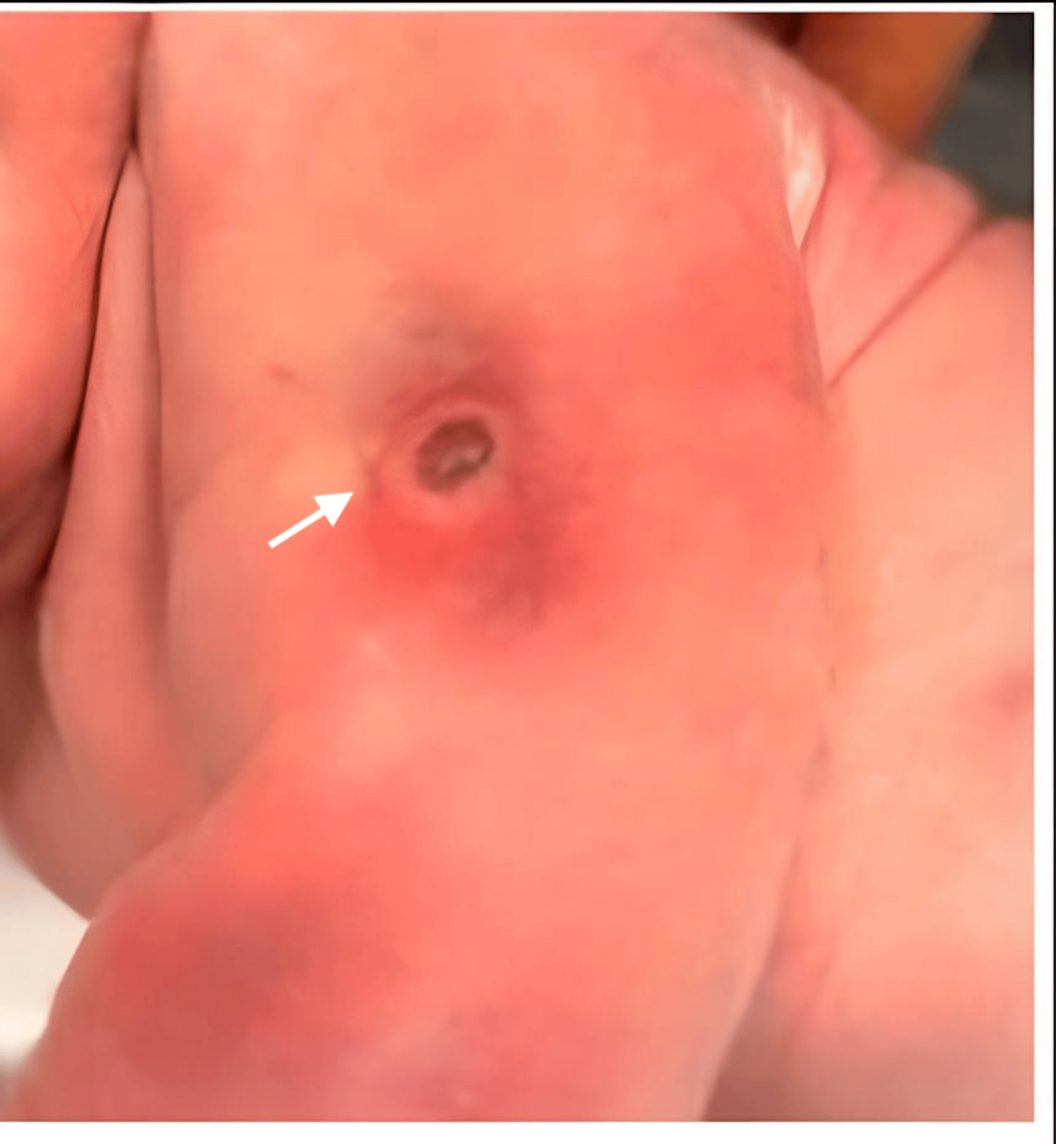
Picture showing a central nodular lesion (20 mm x 15 mm) with an erythematous halo on the left arm of the newborn reflecting necrosis and the surrounding inflammatory reaction

On physical examination, a central nodular lesion with an erythematous halo, elastic consistency, painless on palpation, and apparent adherence to deep planes was observed on the left arm (Figure 1). On the right arm, in a symmetrical location, there was a slight cutaneous depression, soft and without inflammatory signs.

The upper limbs exhibited preserved passive mobility, with wrists in maintained flexion that were reducible upon mobilization, without active extension of the wrist and fingers. The elbows were in slight semi-flexion, with spontaneous movements (Video 1).

**Video 1:** Upon physical examination, the wrists and fingers exhibited no active extension, and the elbows were slightly semi-flexed with some spontaneous movement

**Figure 2:**
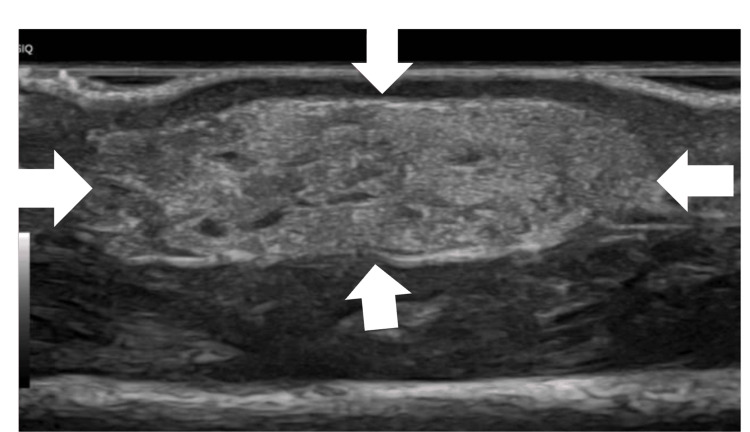
Ultrasound image showing a 14 × 10 × 6 mm hyperechoic, oval lesion, containing small internal cystic components, likely corresponding to a focus of subcutaneous adipose necrosis

Given these findings, laboratory analyses (which included serum calcium levels) and a transfontanellar ultrasound were performed, which revealed no changes. Furthermore, an ultrasound of the soft tissues of the skin lesions showed a hyperechogenic oval structure (14 x 10 x 6 mm) with small internal cystic images, suggesting a probable relationship with a focus of subcutaneous adipose necrosis (Figure 2). In the right arm, ultrasound revealed no significant findings.

After the likely diagnosis of bilateral radial nerve paralysis was made, in a possible case of compression of the radial nerve due to a focus of subcutaneous adipose necrosis, the patient was discharged and scheduled for follow-up in a Neonatology and Pediatric Physical Medicine and Rehabilitation (PM&R) consultation.

Following the hospital assessment by PM&R, a rehabilitation program was commenced on day 15 of life, focusing on neurodevelopmental therapy with an occupational therapist.

During the rehabilitation sessions, the therapist stimulated tactile and proprioceptive sensitivity in the affected limbs by applying tactile stimuli with materials of various textures, sizes, shapes, temperatures, and vibrations and by allowing the child to suck on the thumb of the injured limbs to promote sensorimotor input, oculomotor coordination and central integration of the affected limbs (Video 2).

**Video 2:** Occupational therapy session: sensory stimulation with a vibrating toothbrush

As the child progressed in neurosensory motor development, active mobilization around the body’s midline was encouraged to promote integration and involvement of the affected limbs. Weight-bearing and proprioceptive tasks were introduced in prone positions, while midline activities were performed in both supine and lateral positions.

When in the prone position, supporting the arms enabled full-limb loading and reinforced the stability of the scapular region (Video 3).

**Video 3:** Occupational therapy session: weight-bearing and task stimulation in a prone position

Focusing on the midline facilitated the simultaneous use of both arms in the supine position, fostering bilateral (bimanual) motor coordination (Video 4).

**Video 4:** Occupational therapy session: bilateral (bimanual) motor coordination stimulation

Meanwhile, adopting lateral decubitus positions provided opportunities to work with or against gravity, helping the child uncover and explore the hands in relation to the midline.

As part of the rehabilitation program, parents were educated on the importance of maintaining proper posture for the affected limbs throughout the day with the goal of maintaining joint range of motion and preventing contractures, deformities, and abnormal postural patterns. Parents also learned how to perform smooth passive mobilizations several times a day to avoid joint stiffness and maintain mobility in each impaired joint.

## Discussion

Bilateral isolated radial nerve palsy in newborns is an extremely rare condition. Most cases of radial nerve palsy in newborns are unilateral, with less than 80 reported cases worldwide, and bilateral cases are particularly uncommon [1,3,7,8]. Notably, only 12 bilateral cases have been documented in the literature, as compiled by Carsi et al. [7]. Among these cases, 75% occurred in male newborns, with birth weights ranging from 3.4 kg to 4.7 kg. Prolonged labor was documented in 83% of cases, with 58% involving instrumented deliveries and only two cases resulting from cesarean sections. Primiparity was noted in 66% of cases. Skin lesions were a common finding, present in 75% of the cases, suggesting a potential association with the condition. Recovery times varied significantly, ranging from as short as two weeks to as long as five months.

Isolated radial nerve palsy in newborns is characterized by specific clinical presentations that differentiate it from other types of nerve injuries, such as brachial plexus birth palsy.

Newborns with radial nerve palsy typically present with an inability to extend the wrist and fingers, a condition often referred to as "wrist drop." Despite the weakness in wrist and finger extension, these infants usually have normal function in the shoulder, elbow, and proximal arm muscles, such as the deltoid, biceps, and triceps [2-4,7], unlike those with brachial plexus obstetrical palsy [7,9].

As mentioned previously, the condition is often associated with signs of compression, such as ecchymosis or fat necrosis along the posterolateral brachium, suggesting a compression injury during or before labor [2,4].

We present an additional case of bilateral radial nerve palsy, involving a female newborn delivered at 35 weeks and five days of gestation via cesarean section due to prolonged labor and membrane rupture exceeding 46 hours, following a monitored and uneventful pregnancy with normal prenatal ultrasounds and no evidence of oligohydramnios.

Upon examination, a central nodular lesion with an erythematous halo and elastic consistency was noted on the left arm, while the right arm showed a mild skin depression. Both arms had passive mobility, but active extension of the wrists and fingers was absent. Soft tissue ultrasound revealed subcutaneous adipose necrosis on the left arm, likely causing radial nerve compression, while the right arm showed no significant findings. Laboratory and transfontanellar ultrasound results were normal.

An electromyography exam was requested but deferred, as clinical findings strongly indicated a presumed diagnosis of bilateral radial nerve paralysis.

An early rehabilitation program was then implemented, acknowledging the inherent challenges of treating neonatal patients, including limited patient compliance, difficulty in assessing efficacy, and high family participation [3,9].

The rehabilitation plan adopted a conservative approach centered on neurodevelopmental therapy, led by an occupational therapist. Techniques used during therapy sessions included passive mobilizations, sensory stimulation, bilateral motor integration, and proprioceptive stimulation of the affected limbs.

Recovery of function in the affected limbs was monitored over time primarily through physical observation, allowing for continuous adjustment of interventions.

Gradually, bilateral improvement in active wrist and finger extension was noted, culminating in full recovery within six months of follow-up.

Given the positive clinical progression, it was deemed unnecessary to confirm radial nerve compression via electromyography/nerve conduction studies.

## Conclusions

Differentiating between radial nerve palsy and brachial plexus birth palsy in newborns can be challenging but crucial. A brachial plexus injury typically results in weakened grip strength and limited shoulder and elbow movement, while radial nerve injury specifically affects the wrist and finger extensors. The key distinguishing factor is that shoulder and elbow movement, as well as finger flexion, remain normal in cases of radial nerve injury. Physicians should consider the possibility of isolated radial nerve palsy in a neonate who presents with wrist drop and recognize the differences from brachial plexus palsy during clinical examinations. Once a diagnosis is established, parents can be reassured that bilateral compressive radial neuropathies in neonates, unlike brachial plexus injuries, generally have an excellent prognosis, with most infants recovering fully within six months.

As shown in this case, early rehabilitation interventions can improve recovery outcomes and reduce the risk of musculoskeletal sequelae.
